# Polarization noise places severe constraints on coherence of all-normal dispersion femtosecond supercontinuum generation

**DOI:** 10.1038/s41598-018-24691-7

**Published:** 2018-04-26

**Authors:** Iván Bravo Gonzalo, Rasmus Dybbro Engelsholm, Mads Peter Sørensen, Ole Bang

**Affiliations:** 1 Department of Photonics Engineering, Technical University of Denmark, DK-2800 Kgs. Lyngby, Denmark; 2 Department of Applied Mathematics and Computer Science, Technical University of Denmark, DK-2800 Kgs. Lyngby, Denmark; 3 NKT Photonics A/S, Blokken 84, DK-3460 Birkerød, Denmark

## Abstract

Supercontinuum (SC) generated with all-normal dispersion (ANDi) fibers has been of special interest in recent years due to its potentially superior coherence properties when compared to anomalous dispersion-pumped SC. However, care must be taken in the design of such sources since too long pump pulses and fiber length has been demonstrated to degrade the coherence. To assess the noise performance of ANDi fiber SC generation numerically, a scalar single-polarization model has so far been used, thereby excluding important sources of noise, such as polarization modulational instability (PMI). In this work we numerically study the influence of pump power, pulse length and fiber length on coherence and relative intensity noise (RIN), taking into account both polarization components in a standard ANDi fiber for SC generation pumped at 1064 nm. We demonstrate that the PMI introduces a power dependence not found in a scalar model, which means that even with short ~120 fs pump pulses the coherence of ANDi SC can be degraded at reasonable power levels above ~40 kW. We further demonstrate how the PMI significantly decreases the pump pulse length and fiber length at which the coherence of the ANDi SC is degraded. The numerical predictions are confirmed by RIN measurements of fs-pumped ANDi fiber SC.

## Introduction

Commercially available silica fiber-based and ultra-broadband supercontinuum (SC) sources are typically generated by pumping with high-power picosecond or nanosecond pulses close to the zero-dispersion wavelength (ZDW) in the anomalous dispersion regime of a photonic crystal fiber (PCF)^1,2^. However, the SC source is typically characterized by large intensity fluctuations^2–4^, due to modulation instability (MI) and soliton collisions, which limits their performance for applications in imaging, such as optical coherence tomography (OCT)^5,6^ or coherent anti-Stokes Raman scattering (CARS) spectroscopy^7^. Reduction of the shot-to-shot fluctuations and coherence stabilization can be achieved through various methods, for instance the use of fiber tapers^2,4,8^, seeding with a weak trigger signal^9–11^, or back-seeding part of the SC spectrum^12^. Increasing the repetition rate of the pump laser leads to a noise improvement in spectral domain OCT because of averaging in the spectrometer^13^. An alternative approach to eliminate the influence of noise sensitive effects is to pump a PCF in the normal dispersion regime to avoid MI and soliton collisions. It is however necessary to pump with short enough pump pulses to suppress stimulated Raman scattering (SRS), which is known to be as noisy a process as MI^14,15^. In this way, the bandwidth of the SC is limited not only due to the high dispersion at the pump but also by a constraint to stay away from any ZDW, since noisy SC could be generated when crossing the ZDW^1,14^.

The use of all-normal dispersion (ANDi) PCF fibers, which offer normal dispersion for all the wavelengths covered by the SC^16^, opened up the possibility of potentially coherent broadband SC sources. By pumping with femtosecond pulses, the SC is initiated by self-phase modulation (SPM) and followed by creation of new wavelengths through optical wave breaking (OWB), processes which are known to be coherent^17,18^. Since then, there has been an increasing trend of generating ANDi based SC. Previously, silica fibers with parabolic-like dispersion^19–21^ were pumped at the point of minimum absolute dispersion with high peak powers but the SC extension was limited to 1.5 µm^20^. This issue was overcome with the design of dispersion-flattened germanium doped silica fibers^22,23^ enabling broadening up to 2.2 µm and relaxing the condition of high peak powers^23^. Moreover, ANDi fibers in other materials like soft glasses^24–26^ have been fabricated to push the SC extension towards the mid-infrared, where fiber-based SC sources using chalcogenide fibers are emerging^27–29^.

The superior signal-to-noise ratio performance of ANDi fibers compared to anomalous dispersion pumping was demonstrated experimentally using 390 fs pump pulses by Klimczak *et al*.^30^. However, the SC generation was stabilized with a cladding mode to avoid detrimental effects of SRS due to the long pulse length of the pump^25,30^. A recent numerical study demonstrated the limitations in the coherence of ANDi based SC, pointing out the role of SRS in the process of incoherent dynamics^15^. In general, coherence degradation was found to depend on the relative importance of the mixed parametric-Raman length and the OWB length, which results in a limit that is inverse proportional to the pulse length. For example, for 100 kW peak power and fibers shorter than 1 m complete coherence was found for pump pulses shorter than 700 fs and for pulses shorter than 2 ps complete coherence was found for fibers shorter than 40 cm^15^.

However, in that work^15^ the scalar generalized Schrödinger equation (GNLSE) was used in the modelling, just as in other studies of the coherence of ANDi SC generation^19,20,23,26^. In this way, propagation is assumed in only one axis of the fiber fundamental mode. This means that for example polarization modulational instability (PMI) is neglected, which can be an important source of noise when pumping in the normal dispersion regime^31–33^. In fact, polarization instabilities were experimentally demonstrated in weakly birefringent ANDi fibers^34–36^, which decreases the usability of the ANDi SC source.

It has nevertheless been experimentally demonstrated that PMI, and therefore the noise it introduces, can be suppressed by pumping along a principal axis of a polarization maintaining (PM)-ANDi fiber^35,36^. However, when the polarization of the pump is not along the principal axis of the fiber, PMI-induced noise will be generated, with maximum gain when pumping at 45 degrees^18^. As most of the ANDi SC sources reported are made with non-PM fibers^20–26,30^ an investigation of the polarization properties in non-PM ANDi SC is needed. Therefore, a complete understanding of the decoherence mechanism of the weakly birefringent ANDi based SC, including both SRS and PMI, i.e., both polarizations, is crucial in order to analyze the noise properties and limitations of ANDi SC sources.

In this work we therefore numerically study the coherence and RIN in a standard ANDi fiber for SC generation pumped at 1064 nm, taking into account both polarization components in our model. We demonstrate that the PMI introduces a power dependence not found in a scalar model, which means that even with short ~120 fs pump pulses the coherence of ANDi SC can be degraded at reasonable power levels above ~40 kW. We further demonstrate how the PMI significantly decreases the pump pulse length and fiber length at which the coherence of the ANDi SC is degraded. For example, the scalar model predicts coherence in a 1 m ANDi fiber for pump pulses shorter than about 700 fs, whereas this limit is reduced to only ~120 fs when the second polarization and thus PMI is taken into account, which drastically limits the available pump sources. We further present experimental noise measurements of ANDi based SC pumped with a femtosecond laser, which confirms the numerical predictions.

We want to point out with this study that care must be taken to evaluate the coherence properties of the ANDi SC sources using numerical modelling and that both polarizations should preferably be considered in order to have accurate predictions.

## Results

### Discussion of polarization noise

In our modelling we use the generalized coupled nonlinear Schrödinger equations (CGNLSEs), given in the Methods section. We first reduce our vector model to the single-polarization scalar GNLSE^1,18^ in order to compare to the study done in by Heidt *et al*.^15^. In contrast to that study mode profile dispersion, loss and experimental Raman gain are included in the model to better match the experimental conditions. Note that fiber parameters (pitch and hole-to-pitch ratio) slightly differ.

**Figure 1:**
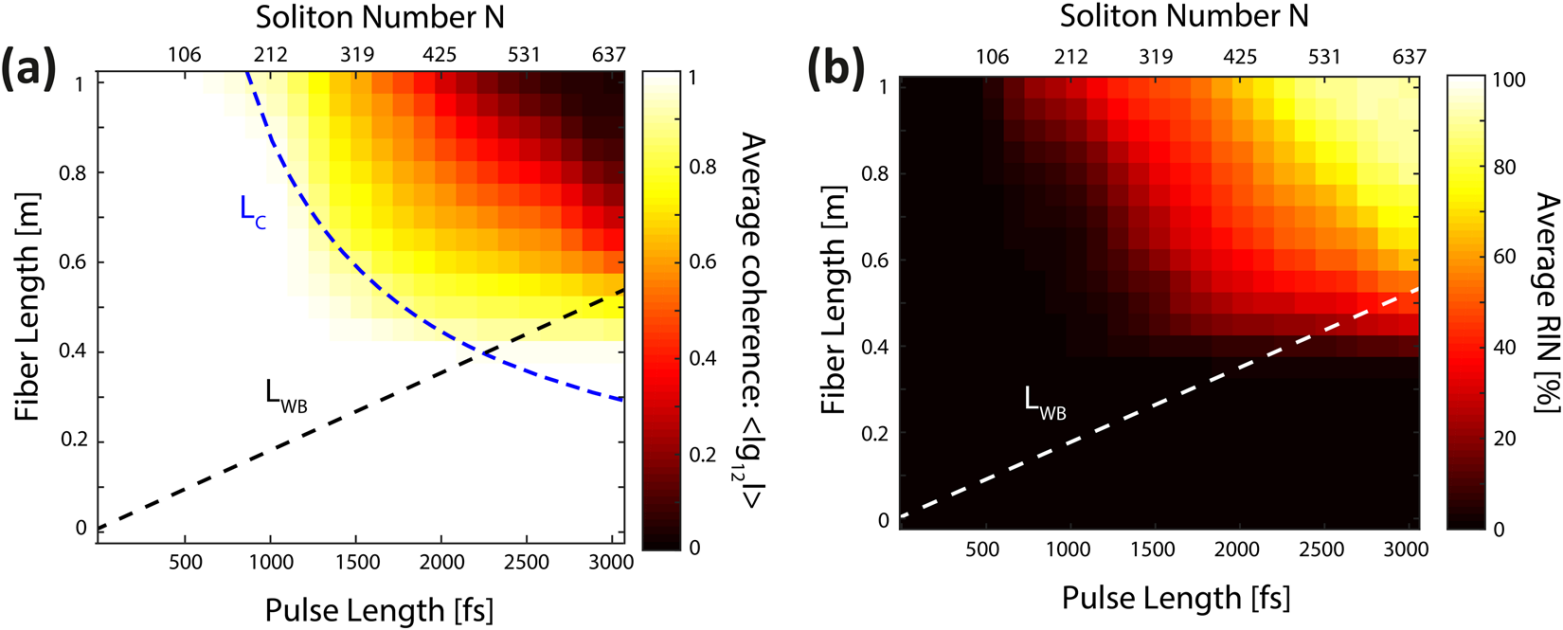
One polarization (scalar model): Spectrally averaged coherence and RIN. (**a**) Average coherence and (**b**) RIN versus pulse length along 1 m of fiber. The peak power is 44 kW, which is the maximum value in the experiments with 0.67 W average power, 80 MHz repetition rate and 170 fs pulse length. The OWB length *L*_*WB*_ and coherence length *L*_*C*_ are given by dashed lines.

For a given fiber length and pump pulse length we calculate the spectrally averaged coherence $\langle |{{g}}_{12}^{(1)}|\rangle$ and spectrally averaged RIN, $\langle {\rm{RIN}}\rangle$ (see the Methods section) to get a single number characterizing the noise and coherence performance. The results are displayed in Fig. 1 for a peak power of 44 kW and pulse lengths from 50 fs to 3 ps along 1 m of fiber every 0.05 m. In evaluating the noise, the important length scales are the OWB length *L*_*WB*_ and the coherence length *L*_*C*_, which for a sech input pulse are given by^15,17^

1 $${L}_{WB}=\sqrt{\frac{3}{2}}\frac{{L}_{D}}{\sqrt{1+{N}^{2}}};\,{L}_{C}\propto \frac{{L}_{R}^{\ast }}{{L}_{WB}}\Rightarrow {L}_{C}=\xi ({\beta }_{2},\gamma ,\,{{\rm{P}}}_{0})\frac{1}{{f}_{R}{{\rm{\Omega }}}_{R}{T}_{0}},$$

where ${L}_{R}^{\ast }$ is the mixed parametric-Raman length evaluated at the angular frequency of the peak Raman gain ${{\rm{\Omega }}}_{R}=2\pi \cdot 13.2\,\,{\rm{THz}}$. In the limit of high pump powers and low absolute dispersion, FWM will tend to suppress the Raman gain and one can obtain the simple expression ${L}_{R}^{\ast }\approx {(1.38{f}_{R}{{\rm{\Omega }}}_{R}\sqrt{\gamma {P}_{0}{\beta }_{2}})}^{-1}$, when taking into account only *β*_2_^15^. The fractional contribution of Raman is${f}_{R}=0.18$. As defined in equation (1), the coherence length corresponds to the fiber length at which the incoherent mixed parametric-Raman process is comparable to the coherent OWB. According to this definition, the coherence length is inversely proportional to the pump pulse length, which follows the trend observed in the simulations^15^. In addition, the function $\xi ({\beta }_{2},\gamma ,{P}_{0})$ has units of length and it is introduced to fit the coherence length to the edge of the region where $\langle |{{g}}_{12}^{(1)}|\rangle =0.9$ in the plot of the average coherence as a function of pulse length^15^. Therefore, the coherence length is an analytical estimate of the propagation distance at which the coherence degradation starts in the simulations for a given pulse length, and it is introduced here to facilitate the discussions of the ANDi SC coherence. The soliton number *N*, the dispersion length *L*_*D*_, and the nonlinear length *L*_*N*_, are given by^1^

2 $$N=\sqrt{\frac{{L}_{D}}{{L}_{N}}};\,{L}_{D}=\frac{{T}_{0}^{2}}{|{\beta }_{2}|};\,{L}_{N}=\frac{1}{\gamma {P}_{0}}.$$

The results presented in Fig. 1 recover the same qualitative behavior obtained by Heidt *et al*.^15^, and show that the coherence is maintained and RIN is low for the first 1 m of the fiber for pulse lengths shorter than around 1 ps. Due to the simpler model and slightly different fiber this limit was found to be around 0.7 ps in the previous study by Heidt *et al*.^15^.

In general, when fiber lengths longer than the coherence length are used, the generated SC is affected by Raman noise and will have reduced coherence. When the fiber length is longer than the OWB length but shorter than the coherence length, the SC is completely developed before Raman lines are generated and thus the broadest possible SC is generated coherently. This occurs for pulse lengths shorter than around 2.2 ps, as seen in Fig. 1a. Using pump pulses longer than 2.2 ps Raman noise will be generated before OWB and thus only a narrow coherent SC can be generated. The dynamical evolution of the noise is studied in more detail by Heidt *et al*.^15^. In addition to the coherence, we also consider the spectrally averaged RIN shown in Fig. 1b, which is seen to follow a similar trend as the coherence, not surprisingly, considering the definition used. Therefore, we can verify qualitatively the results obtained by Heidt *et al*.

**Figure 2:**
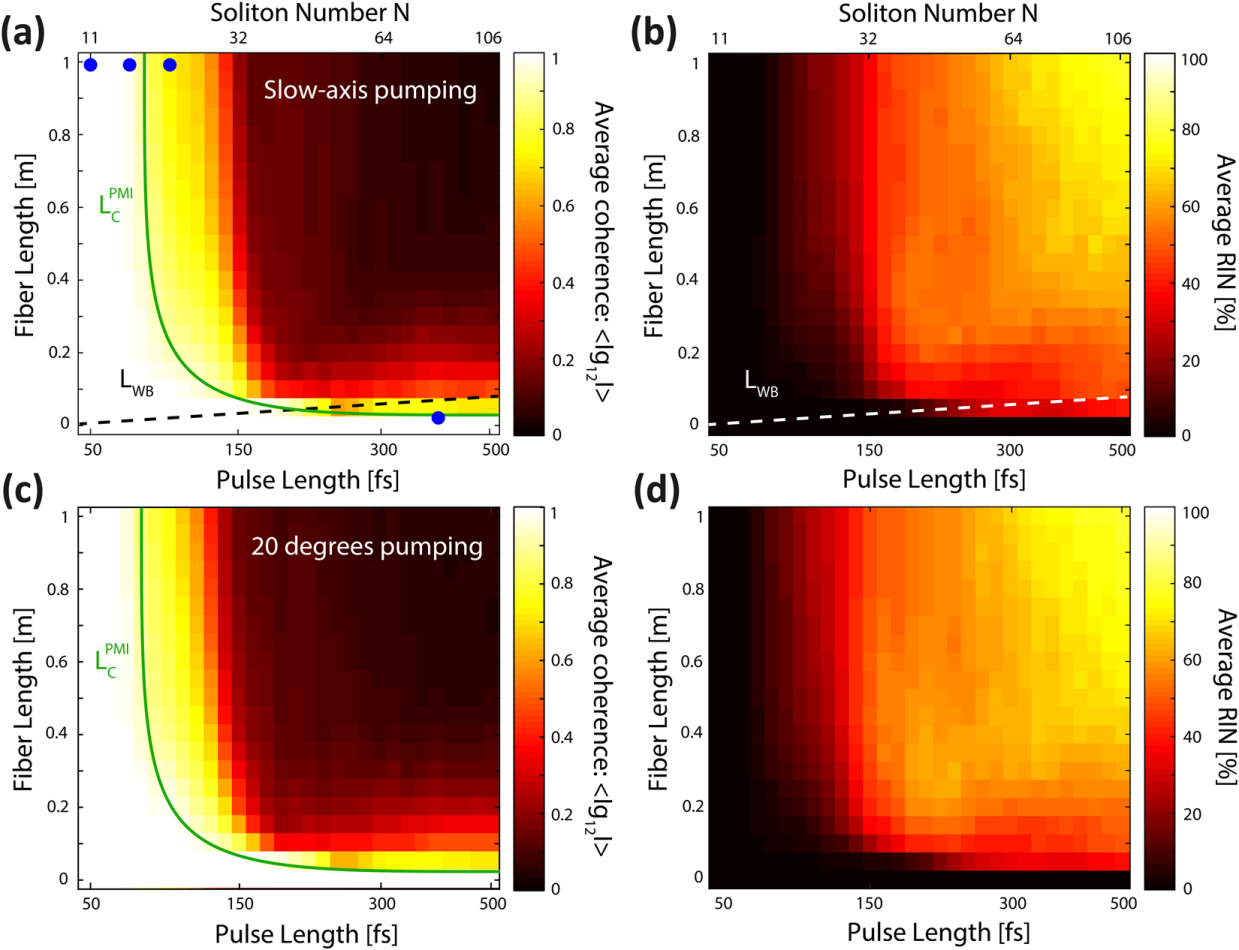
Two polarizations (vector model): Spectrally averaged coherence and RIN. Average coherence and RIN calculated with input polarization along the slow-axis (**a,b**) and with a 20 degrees angle with respect to the slow-axis (**c,d**) versus pulse length along 1 m of fiber. The peak power is 44 kW as in Fig. 1. ${L}_{C}^{PMI}$(green line) indicates the maximum propagation distance for which the average coherence is higher than 0.9.

After analyzing and verifying the numerical implementation obtained with the scalar approach, we now study the effect of including the second polarization of the fundamental mode in the model by solving the CGNLSEs. Fiber birefringence is also included as the unintentional birefringence measured in the ANDi fiber (see the Methods section). All the parameters are the same as for the scalar model except for the pulse lengths (from 50 fs to 500 fs), which are chosen within the coherent regime in Fig. 1, where the noise from SRS has a minor contribution. Figure 2 shows the spectrally averaged coherence and RIN for two different input polarizations, one along the slow-axis (Fig. 2a,b), and the other at a 20 degrees angle with respect to the slow-axis of the fiber (Fig. 2c,d).

In contrast to the scalar model, the vector simulations show that degradation of the phase and intensity stability starts already for much shorter pump pulses and much shorter fiber lengths. The PMI influenced coherence length, ${L}_{C}^{PMI}$, shown in Fig. 2a and c, indicates the edge of the region where the coherence of the SC is maintained, which we define as when the coherence is higher than 0.9. In the limit of long pulse lengths, it goes towards the analytical PMI length, which for slow-axis pumping is given by ${L}_{PMI}=3/2\gamma {P}_{0}=1\,{\rm{mm}}$ for the parameters used in Fig. 2a.

Figure 2 shows that the good noise properties, up to 1 ps after 1 m of propagation predicted by the scalar model in Fig. 1, are lost already at about 120 fs with the vector model for both slow-axis and 20 degrees pumping. The results further show that coherent SC with longer pulse lengths than 150 fs can only be achieved with very short fiber lengths, shorter than about 5 cm, again for both pump configurations. Let us consider the dependence of the input polarization on PMI in our case: Since we are pumping in the normal dispersion regime there is a critical power for PMI when pumping along the fast-axis^18^, which is ${P}_{cr}^{fast}=3\pi \,{\rm{\Delta }}n/{\lambda }_{p}\gamma =3.2\,\,{\rm{kW}}{\rm{.}}$ So when pumping with a peak power of 44 kW and increasing the angle relative to the slow-axis, the power in the slow-axis is reduced, resulting in a reduced PMI gain, and the power in the fast-axis is increased, achieving the threshold at an angle of 15.6 degrees. Thus, for 20 degrees pumping there is PMI from the fast-axis in addition to the PMI from the slow-axis with reduced gain. This fact means that we see only a minor difference in the coherence and RIN between the two configurations seen in Fig. 2a–d, respectively.

**Figure 3:**
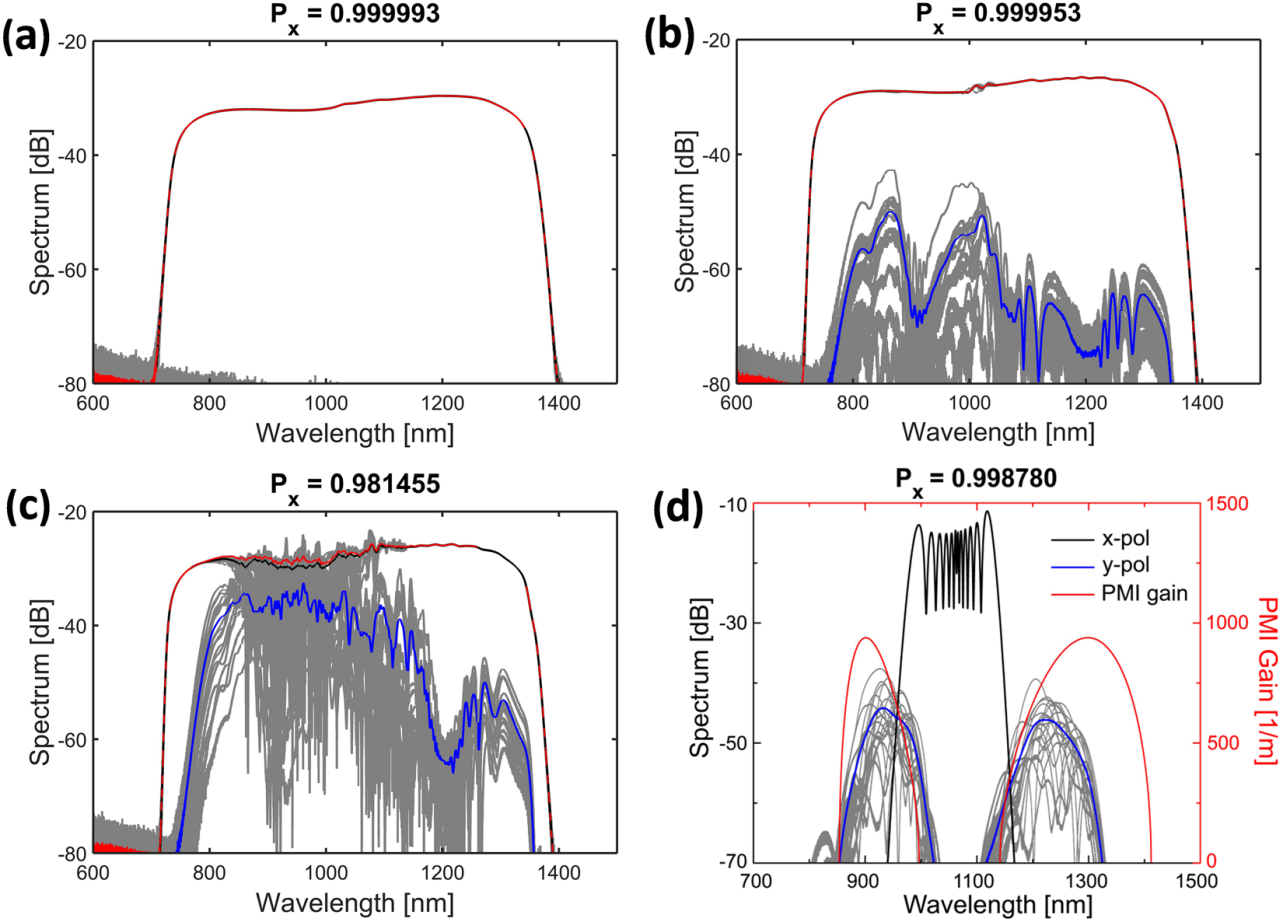
Simulated SC for both polarizations. Mean total spectrum (red), mean x-polarization (black), mean y-polarization (blue). The spectral fluctuations are shown in grey for the x and y-polarization. (**a**) 50 fs, (**b**) 100 fs, and (**c**) 120 fs pump pulse at 1 m and (**d**) 400 fs at 25 mm, corresponding to the blue dots in Fig. 2. The fractional power in the x-polarized field at 1 m is given by P_x_.

In Fig. 3 we consider in more detail the 4 cases marked in Fig. 2a with blue dots for slow-axis pumping, to see the spectral distribution of the power in the two polarizations. Pumping with the shortest pulses, 50 fs and 100 fs, almost no transfer of energy between the polarizations occurs (the x-polarization power is identical to the total power), resulting in good coherence over 1 m. However, more energy is transferred between polarizations already at 120 fs and coherence degradation takes place. Furthermore, while not shown here, we observe that power transfer between the polarization states saturates for long enough propagation distances. This behavior agrees well with previous experiments carried out with the same ANDi fiber^34^, where the power in the slow- and fast-polarization was measured for pumping along the slow-axis.

From Fig. 3, we can identify PMI as the mechanism that leads to coherence degradation. The interaction between the polarizations due to PMI results not only in transfer of power but also noise between them, which seems to be the main effect responsible for coherence degradation. For 100 fs, amplification of the noise floor through PMI in the y-polarization takes place, similarly to how scalar MI amplifies noise in anomalous dispersion pumped SC^1,37^. This results in a very noisy but low power SC in that polarization (Fig. 3b). However, as most of the power is still in the x-polarization, whose noise properties remain good, the total relative noise is low. Once the PMI gain is sufficiently high to lead to stronger interaction between polarizations, such as for the 120 fs case, the power in the noisy y-polarization is higher and contributes more to the total relative noise. This together with the transfer of noise to the x-polarization will result in a noisier total SC, as shown in Fig. 3c. Therefore, stronger interaction between the polarizations leads to a noisier total SC.

To confirm the presence of PMI we plot in Fig. 3d (red curve) the theoretical gain profile, which shows the well-known two sidebands around the pump for slow-axis pumping, with a maximum gain and wavelength detuning being determined only by the input power when the fiber parameters are fixed (dispersion, nonlinearity and birefringence)^18,31–33^. The simulation in Fig. 3d for a 400 fs pump pulse shows agreement with the sidebands calculated theoretically. Higher order dispersion terms and Raman are not included in the simple analytical small-signal gain PMI model^18^, resulting in the observed deviation between simulation and theory. As we shall see in the experimental section, the characteristic PMI sidebands are also observed experimentally.

In order to gain more insight into the polarization noise and with the aim of comparing the modelling to the experiments, simulations with different input powers were performed for a fixed fiber length of 0.5 m. We also need to consider the effect of a chirp on the input pulse, because our 80 MHz pump laser emits chirped pulses with a FWHM bandwidth of the intensity profile of ${\rm{\Delta }}{\lambda }_{FWHM}=16.6\,{\rm{nm}}$. Assuming a quadratic chirp of an input 170 fs (${T}_{0}=96.4\,{\rm{fs}}$) sech pulse $U(0,{\rm{T}})$ = sech $(T/{T}_{0})\exp (\,-\,iC({T}^{2}/{{T}_{0}}^{2}))$, we estimate the chirp parameter to be $C=\sqrt{{({\rm{\Delta }}{\lambda }_{FWHM}/{\rm{\Delta }}{\lambda }_{FWHM}^{TL})}^{2}-\,1}=2.15$, where ${\rm{\Delta }}{\lambda }_{FWHM}^{TL}$ is the transform limited FWHM bandwidth of the intensity profile. Calculations are done with both chirped and un-chirped input pulses.

**Figure 4:**
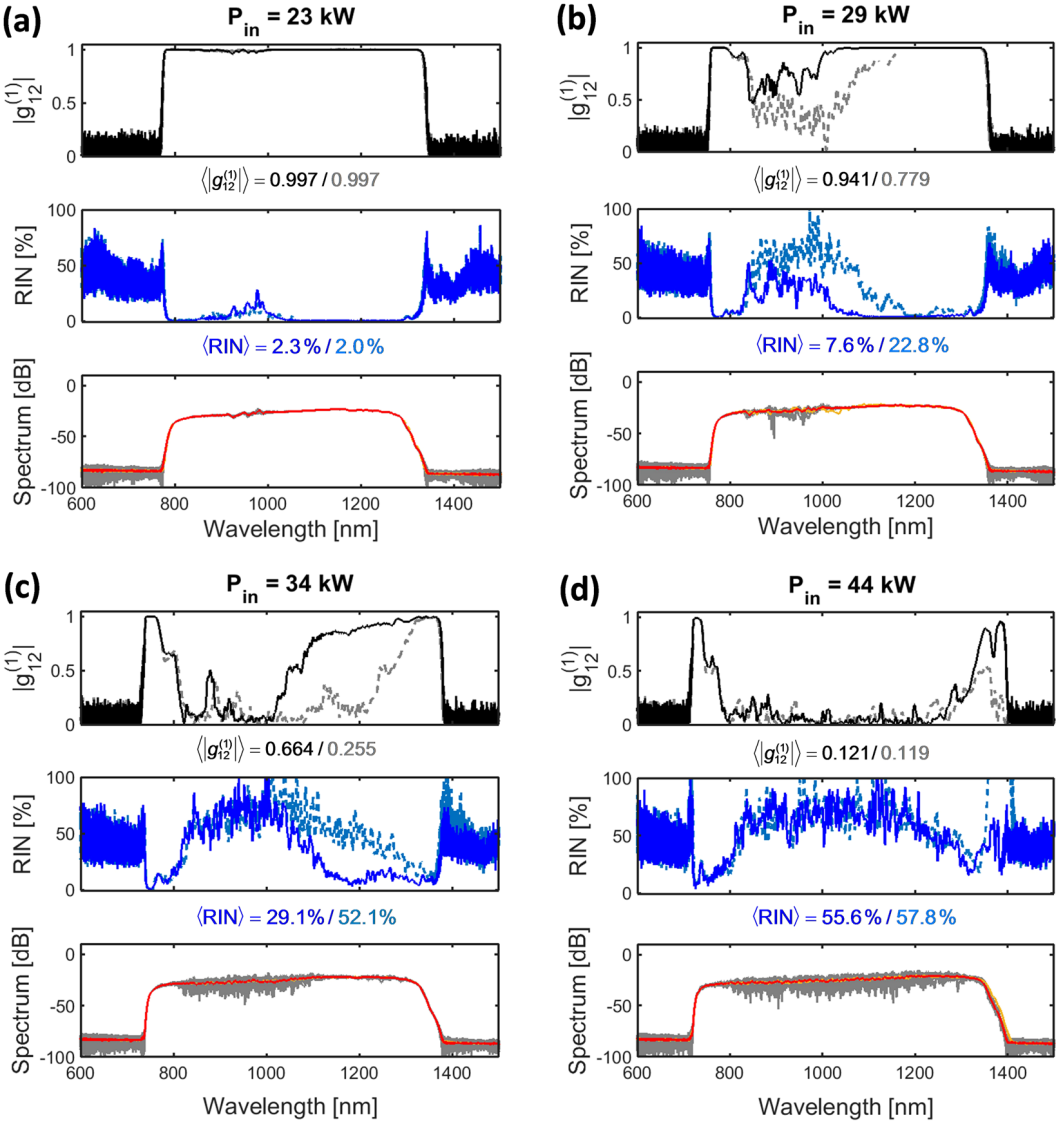
Dependence of RIN and coherence on input peak power for 170 fs pumping. Calculated mean SC spectra (red), RIN (blue) and $|{{g}}_{12}^{(1)}|$ (black) vs wavelength pumping with 170 fs and 20 degrees respect to slow-axis at 0.5 m using input peak powers (and corresponding output average powers in the experiments) (**a**) 23 kW (0.36 W), (**b**) 29 kW (0.45 W), (**c**) 34 kW (0.53 W) and (**d**) 44 kW (0.67 W). Results for chirped (un-chirped) pump pulses are shown as full (lighter dashed) curves.

Figure 4 shows the evolution of the spectrum and the spectral profiles of the RIN and coherence as a function of the input power for a 170 fs pump with input polarization at 20 degrees. As expected, at a low input power of 23 kW (Fig. 4a), a SC with low RIN ($\langle {\rm{RIN}}\rangle =2.3\, \%$) and high coherence ($\langle |{{g}}_{12}^{(1)}|\rangle =0.997$) across the whole profile is generated. This is because the PMI gain is not sufficiently high to amplify noise from the other polarization within the chosen fiber length. Increasing the input power (Fig. 4b,c,d) to achieve a broader spectrum the PMI gain is increased, resulting in lower coherence and higher RIN. Already at 23 kW a weak and narrow noise band appears below the pump wavelength (Fig. 4a). At higher pump powers the strength and bandwidth of the noise increases (Fig. 4b,c) and at 44 kW coherence is lost across the whole bandwidth of the spectrum, except for in a very narrow region around the edges (Fig. 4d).

The results for an un-chirped input pulse are shown as lighter dashed lines and they show the same general behavior, but interestingly demonstrate that the coherence and RIN performance is improved when pumping with a chirped pulse, while the spectrum remains unchanged. The average RIN and coherence for 29 kW is for example, $\langle {\rm{RIN}}\rangle =22.8\, \%$ and $\langle |{{g}}_{12}^{(1)}|\rangle =0.779$ for the un-chirped pump, and improved to 7.6% and 0.941 for a chirped pump, respectively (Fig. 4b).

### Supercontinuum and relative intensity noise measurements

**Figure 5:**
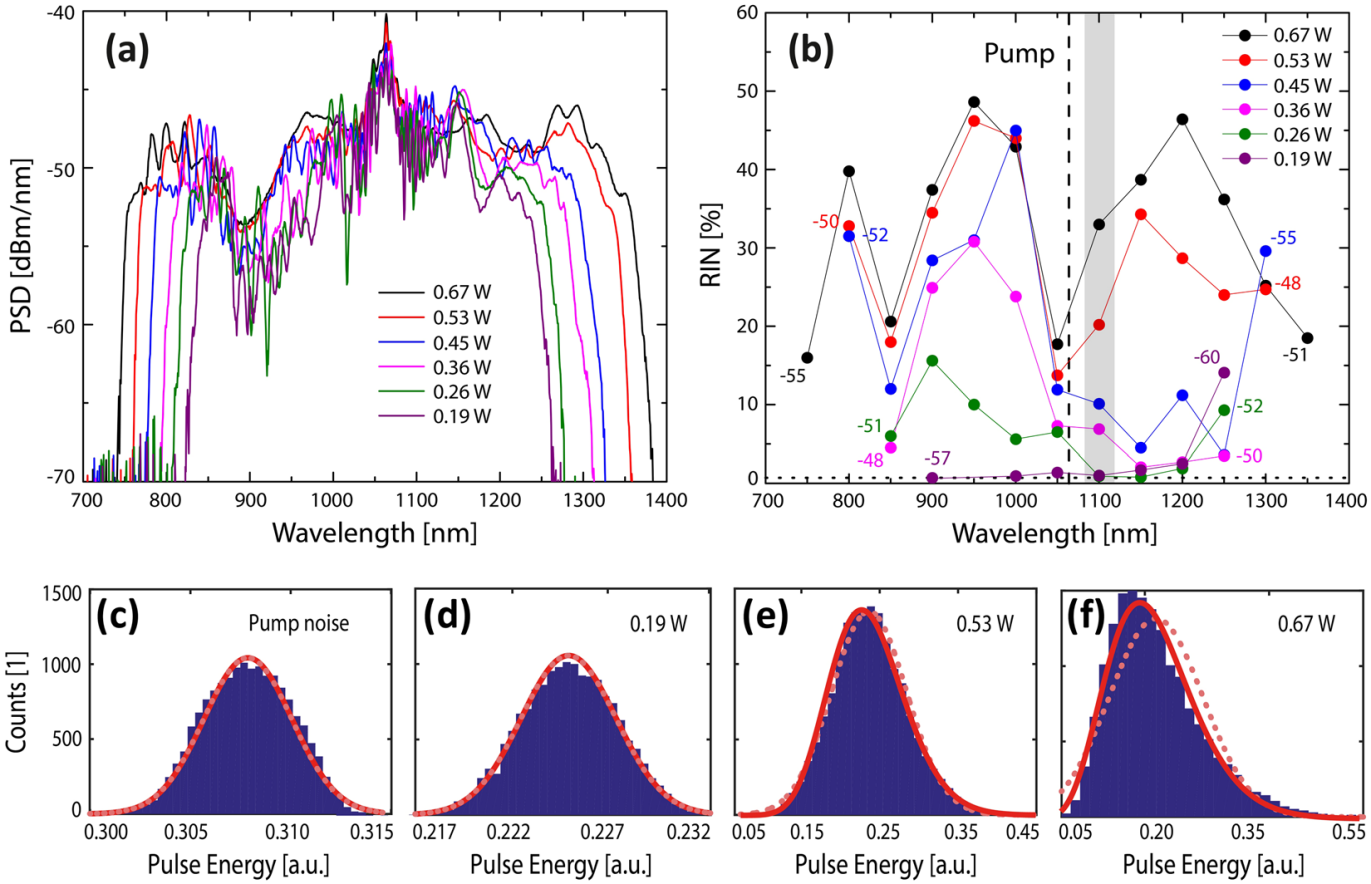
Measured supercontinuum and RIN vs power for a 170 fs pump and 0.5 m long ANDi fiber. SC spectra (**a**) and corresponding RIN spectra (**b**) for different output average powers. The dotted horizontal line in (**b**) is the noise of the pump laser, 0.71%, and the numbers indicate the power level in dBm/nm. Histograms of the pulse energy and corresponding Gamma (full red) and Gaussian (dotted light red) fit of the 1064 nm pump (**c**) and a 10 nm band of the SC at 1100 nm (grey bar) for output average powers of (**d**) 0.19 W, (**e**) 0.53 W and (**f**) 0.67 W.

**Figure 6:**
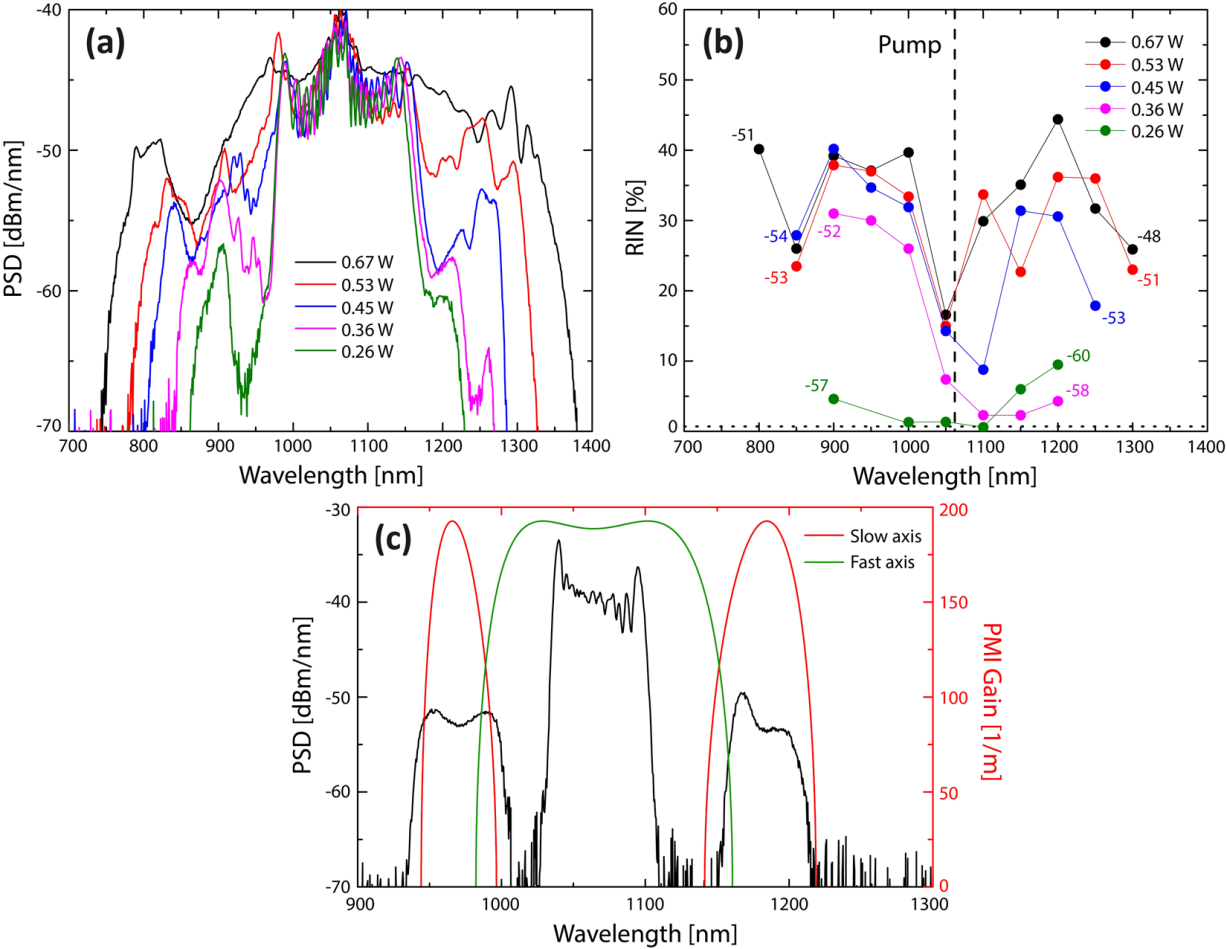
Measured supercontinuum and RIN vs power for a 235 fs pump and 0.5 m long ANDi fiber. SC spectra (**a**) and corresponding RIN spectra (**b**) for different output average powers (numbers in (**b**) indicate the power level in dBm/nm). The dotted horizontal line in (**b**) is the noise of the pump laser, 0.67%, and the numbers indicate the corresponding power levels in dBm/nm. (**c**) SC spectrum with 0.4 W output power for a 540 fs pump pulse (black) and corresponding theoretical PMI gain for slow-axis (red) and fast-axis (green) pumping.

The numerical study presented above shows that the coherence is degraded and noise increases when increasing the input power for a given fiber length due to the presence of PMI. These results are confirmed in this section, where the noise of a femtosecond pumped ANDi SC is experimentally measured for two pump pulse length configurations, 170 fs (Fig. 5) and 235 fs (Fig. 6). The details of the experimental setup and the method used to measure the RIN as a function of wavelength are given in the Methods section.

Figures 5a and 6a show the SC generated with 170 fs and 235 fs, respectively, for different power levels ranging from 0.19 W to 0.67 W using 0.5 m of the ANDi fiber. For the SC generated at each power level, the corresponding RIN, shown in Figs 5b and 6b, is measured every 50 nm with 10 nm bandwidth filters across the SC spectrum. Let us focus on the general trends:

As expected from the numerical modelling, the SC generated at the lowest pump powers has the lowest noise, for both pump pulse configurations, which is at the same level as the 0.71% RIN of the pump laser (black dotted line), indicating that there is negligible noise added by the nonlinear SC processes. Increasing the pump power is seen to generate a broader SC and lead to an increase of RIN as also found numerically. In terms of the spectral RIN profile, the noise is seen to start at wavelengths below the pump for both pump pulse lengths, again as found numerically in Fig. 4. For example, pumping with 170 fs low RIN of less than 10% can be obtained for wavelengths above the pump for output powers up to around 0.45 W, but the RIN is already high at shorter wavelengths below the pump for an output power of 0.26 W.

Another common feature for both pulse lengths is that at higher pump powers the RIN around the pump wavelength drops to very low values, which was not found in the simulations in Fig. 4. This could be explained by pump light propagating in the cladding, which has been demonstrated to lower the noise around that wavelength^30^.

Examples of the filtered pulse energy histograms from which the RIN value is calculated is shown for the un-filtered 1064 nm pump in Fig. 5c and for a 10 nm band around 1100 nm for three different power levels in Fig. 5d–f. The fitted gamma distributions, from which the RIN in Fig. 5b was calculated (see the Methods section) are shown as solid red curves, whereas a fitted Gaussian is shown as a red dotted curve. The histograms show a gradual increase of the FWHM of the Gamma distribution (of the noise) and a transition from a Gaussian distribution for low powers (0.19 W) to a more skewed distribution for higher powers (0.67 W). To the best of our knowledge this observation is new and in fact non-trivial, because it says that the noise statistics of a fs ANDi SC follow the same trends as those presented for MI driven SC pumped in the anomalous dispersion regime reported before^37,38^, further indicating a connection between MI and PMI, even if the soliton dynamics is absent in ANDi SC generation.

To demonstrate experimentally that the ANDi SC is initiated by PMI we increase the pulse length to 540 fs and decrease the power to give an output average power of 0.4 W, corresponding to 8.5 kW input peak power if one neglects loss. The spectrum shown in Fig. 6c clearly reveals the separated PMI gain bands obtained for pumping along the slow-axis, coinciding with the analytically predicted PMI gain bands (red curve). The presence of the single central gain band obtained when pumping along the fast axis (green curve) cannot be determined and thus we cannot say whether we are pumping along an optical axis. Most probably we are not and both types of PMI gain bands are present. In any case the experiments verify the presence of PMI in the ANDi SC generation.

### Noise comparison between simulations and measurements

We performed an ensemble of 20 simulations using the experimental conditions, with certain approximations: a hyperbolic sech pulse was assumed as input pulse, and positive chirp was added according to the measured bandwidth. The fiber dispersion and nonlinearity used in the simulation were calculated with COMSOL and might thus differ slightly from the actual fiber dispersion and nonlinearity. The loss was included in the simulations (see the Methods section) although its influence is negligible since the fiber length was only 0.5 m. Coupled-in power was estimated based on measured output power, taking into account the fiber output facet transmission, but neglecting loss in the fiber.

**Figure 7:**
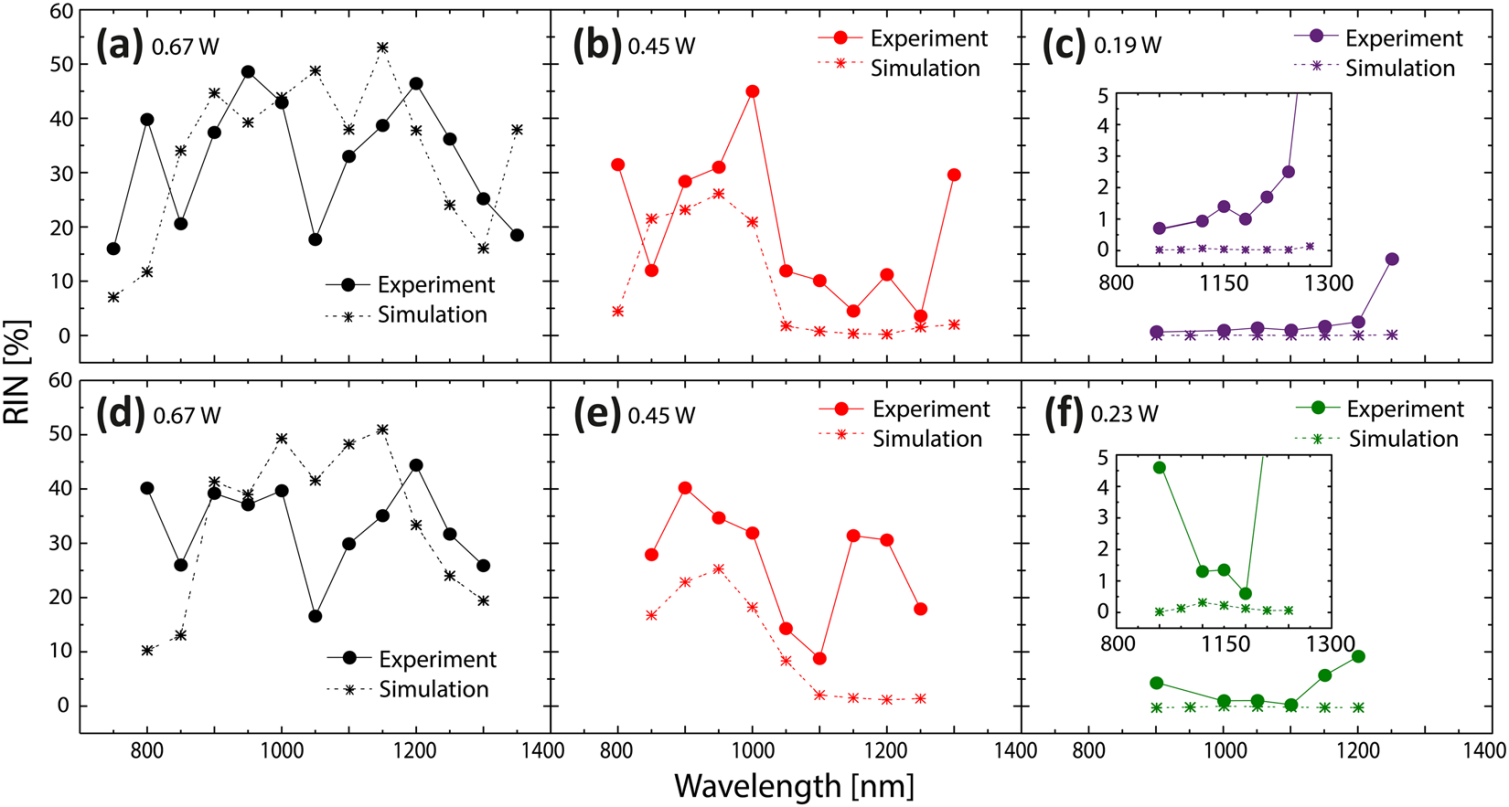
Comparison between measured and calculated RIN. Measured (circle + solid line) and calculated (star + dashed line) RIN vs wavelength for (**a**–**c**) 170 fs and (**d**–**f**) 235 fs pump pulses for three output average power levels. Insets in (**c**) and (**f**) show a close-up for better comparison of the measured and calculated RIN.

Figure 7 shows the measured and calculated spectral RIN profiles for the two different pump pulse lengths used in the experiment, for 3 different average output power levels. The calculated RIN was filtered every 50 nm over 10 nm bandwidth to match simulations to the experiments, since noise is bandwidth dependent^39^. Because of filtering (averaging) over 10 nm, the noise is lower than the one calculated in Fig. 4, where the RIN was not wavelength filtered.

The general behavior of the measured RIN is reproduced by the simulations for both pump pulse lengths, showing the same general power and wavelength dependence as also discussed in connection with Figs 5 and 6 above, i.e., that the noise increases with power (for instance, Fig. 7a,b,c) and starts at wavelengths below the pump (Fig. 7b,e). Taking into account the uncertainty in the pump power level and pump profile, the quantitative level is quite well reproduced, except at the pump at high power and in some cases close to the edges. The edge points are highly influenced by the fact that there is very little power (generally below −50 dBm/nm as seen in Figs 5 and 6) and should probably not be considered. The RIN close to the pump in Fig. 7a,b could be highly influenced by pump light in the cladding as discussed above^30^.

One observation is that the calculated RIN is usually lower than in the experiments. For the 170 fs case with the lowest power (0.19 W), the RIN is almost zero in the simulations in contrast to the experiment as shown in Fig. 7c. This can be explained by the intensity fluctuations of the pump laser (0.71%), which sets the lower limit for the experiment. While not shown here, simulations performed with 1% variation in the pump intensity reproduce very well this lower limit behavior.

## Conclusion

Design of broadband low-noise SC sources using ANDI fibers requires a good understanding of the phenomena involved in the process in order to avoid any possible noise sources. The coherence is usually calculated with the GNLSE following a scalar approach, which ignores polarization effects, such as PMI. With the use of the vector GNLSE, the two polarizations of the fundamental mode are included and thus the noise arising from PMI and other polarization processes can be studied in order to optimize the noise performance of the SC source.

We have investigated numerically the coherence and noise of SC in a standard ANDi fiber with the CGNLSE, explaining the mechanism of polarization noise in this weakly birefringent fiber pumped with femtosecond pulses. Vector propagation simulations unveil PMI driven noise in ANDi SC, which is hidden when doing scalar simulations. Polarization modulational instability redistributes the power between the two polarizations leading to intensity and phase fluctuations. The polarization noise depends not only on the pump parameters, such as pulse length, power and polarization orientation, but also on the fiber length. Comparing to the single-polarization case, the fiber length at which the ANDi SC is fully coherent (coherence length *L*_*C*_ defined by Heidt *et al*.^15^) is drastically reduced to the PMI influenced coherence length ${L}_{C}^{PMI}$, and thus fully coherent SC can in a 1 m ANDi fiber for example only be achieved with pulses shorter than around 120 fs, in contrast to the 1 ps obtained with the scalar model.

The optimum conditions for designing a low-noise ANDi SC source are thus not trivial and depend strongly on both the nonlinear fiber properties and the pump configuration, in particular also on their polarization properties. From the results presented here, we can conclude that there are several regions of operation for a given fiber type and pump. According to the obtained numerical results, broadband fully coherent ANDi SC can be easily achieved in general with short fiber lengths and short femtosecond pump pulses. The crossing point of *L*_*WB*_ and ${L}_{C}^{PMI}$ in Fig. 2 sets the maximum pump pulse length to about 180 fs for low noise SC operation in which optical wave breaking is fully used to generate the broadest possible spectrum. Above 180 fs PMI will generate noise before the broadest ANDi SC is achieved. Shorter pump pulses than 180 fs can be used to pump longer fibers without coherence degradation, for instance, pumping with 50 fs the decoherence will start after more than 1 m. In this case, the upper limit for the fiber length is set by the point at which the SRS starts to generate noise. Another option to achieve broadband coherent ANDi SC would be to use PM-ANDi fibers as previously proposed^35,36^, but PMI-induced noise will be suppressed only when pumping along one of the principal axes of the fiber^18^. Therefore, a good control of the input polarization for coherent ANDi SC with PM fibers would be required.

The numerical results obtained with the CGNLSE were confirmed experimentally by pumping a 0.5 m long commercially available ANDi fiber with a femtosecond laser. The results of the measured RIN of the ANDi SC for two pump pulse lengths verified the power and wavelength dependence of the SC noise. Polarization modulation instability gain sidebands were also observed using 540 fs pump pulses, verifying that PMI is the decoherence mechanism involved in the SC generation in the weakly birefringent ANDi fiber under study.

## Methods

### Numerical model

In our study we use the well-known CGNLSEs, written in terms of circular polarization components, in which the two orthogonal polarizations of the fundamental mode are included^18,40–42^

3 $$\begin{array}{rcl}\frac{\partial {\tilde{C}}_{1}({\rm{\Omega }},z)}{\partial z} & = & i\frac{{\rm{\Delta }}{\beta }_{0}}{2}{\tilde{C}}_{2}({\rm{\Omega }},z)+i[\beta (\omega )-[\beta ({\omega }_{0})+{\beta }_{1}({\omega }_{p}){\rm{\Omega }}]]{\tilde{C}}_{1}({\rm{\Omega }},z)-\frac{\alpha (\omega )}{2}{\tilde{C}}_{1}({\rm{\Omega }},z)\\  &  & +i\bar{\gamma }(\omega )(1+\frac{{\rm{\Omega }}}{{\omega }_{0}})\cdot {\rm{F}}\{(1-{f}_{R}){C}_{1}(t,z)[\frac{2}{3}|{C}_{1}(t,z){|}^{2}+\frac{4}{3}|{C}_{2}(t,z){|}^{2}]\\  &  & +{f}_{R}{C}_{1}(t,z)\cdot {{\rm{F}}}^{-1}\{{\tilde{h}}_{R}({\rm{\Omega }})\cdot {\rm{F}}\{|{C}_{1}(t,z){|}^{2}+|{C}_{2}(t,z){|}^{2}\}\}\},\end{array}$$

4 $$\begin{array}{rcl}\frac{\partial {\tilde{C}}_{2}({\rm{\Omega }},z)}{\partial z} & = & i\frac{\Delta {\beta }_{0}}{2}{\tilde{C}}_{1}({\rm{\Omega }},z)+i[\beta (\omega )-[\beta ({\omega }_{0})+{\beta }_{1}({\omega }_{p}){\rm{\Omega }}]]{\tilde{C}}_{2}({\rm{\Omega }},z)-\frac{\alpha (\omega )}{2}{\tilde{C}}_{2}({\rm{\Omega }},z)\\  &  & +i\bar{\gamma }(\omega )(1+\frac{{\rm{\Omega }}}{{\omega }_{0}})\cdot {\rm{F}}\{(1-{f}_{R}){C}_{2}(t,z)[\frac{2}{3}{|{C}_{2}(t,z)|}^{2}+\frac{4}{3}{|{C}_{1}(t,z)|}^{2}]\,\\  &  & +{f}_{R}{C}_{2}(t,z){{\rm{F}}}^{-1}\{{\tilde{h}}_{R}({\rm{\Omega }})\cdot {\rm{F}}\{|{C}_{2}(t,z){|}^{2}+|{C}_{1}(t,z){|}^{2}\}\}\},\end{array}$$

where ${{\rm{C}}}_{1,2}(t,z)$ are the pseudo-field envelopes of the two circular polarization components in the time domain, and their Fourier transforms are given by

5 $${\tilde{C}}_{1,2}({\rm{\Omega }},z)={[\frac{{A}_{eff}(\omega )}{{A}_{eff}({\omega }_{0})}]}^{-\frac{1}{4}}{\tilde{A}}_{1,2}({\rm{\Omega }},z),$$

where ${\tilde{A}}_{1,2}({\rm{\Omega }},z)$ are the field envelopes in the frequency domain of the two circular polarization components, Ω = ω − *ω*_0_, and ${A}_{eff}(\omega )$ is the effective area. The transformation to the pseudo-field envelopes in equation (5) is done to include mode profile dispersion^43^. In this way the photon number is conserved when the loss is set to zero. In this version of CGNLSEs the nonlinear parameter is modified and given by

6 $$\bar{\gamma }(\omega )=\frac{{\omega }_{0}{n}_{2}{n}_{eff}({\omega }_{0})}{c{n}_{eff}(\omega )\sqrt{{A}_{eff}(\omega ){A}_{eff}({\omega }_{0})}},$$

where ${n}_{2}=2.66\times {10}^{-20}{m}^{2}/W$ is the value of the nonlinear refractive index of silica, and ${n}_{eff}(\omega )$ is the effective refractive index of the fundamental mode calculated with COMSOL Multiphysics. Equations (3) and (4) include the full dispersion, where *ω*_0_ is the central frequency of the numerical frequency domain and *ω*_*p*_ is the pump frequency. The total loss $\alpha (\omega )={\alpha }_{m}(\omega )+\,{\alpha }_{c}(\omega )$ is also included as the contribution of the material loss, ${\alpha }_{m}(\omega )$, of silica^44^ and the confinement loss, ${\alpha }_{c}(\omega )$, which is calculated from the imaginary part of the effective refractive index obtained with COMSOL Multiphysics. Finally, the measured Raman response function of a silica fiber in the frequency domain is included as ${\tilde{h}}_{R}({\rm{\Omega }})$, and ${f}_{R}=0.18$ is the fraction of the Raman contribution to the nonlinear polarization.

For the case under study, in which a weakly birefringent fiber is considered, the nonlinear parameter and dispersion is assumed to be the same for the two polarizations^18,34,41,42^. The birefringence of the fiber is added through the phase mismatch between the two polarization modes given by $${\rm{\Delta }}{\beta }_{0}={\beta }_{0,x}-{\beta }_{0,y}=[{n}_{eff,x}({{\rm{\omega }}}_{0})-$$$${n}_{eff,y}({{\rm{\omega }}}_{0})]\,{{\rm{\omega }}}_{0}/c={\rm{\Delta }}n\,{{\rm{\omega }}}_{0}/c$$, where $${\rm{\Delta }}n=1.3\times {10}^{-5}$$ was measured in^34^ and specified by the supplier, NKT Photonics. Note that the group velocity mismatch between the axes $$({\rm{\Delta }}{\beta }_{1}=0)$$ can be ignored in the CGNLSEs for relatively low-birefringent fibers^18,34,41,42^, and it was neglected in our model.

The input conditions to equations (3) and (4) are circularly field envelopes in the time domain given by $${A}_{1,2}(T,0)=$$$${U}_{z=0}\exp (\pm i\theta )/\sqrt{2}$$, where the input field envelope is a chirped sech pulse $${U}_{z=0}$$ = sech $$(T/{T}_{0})\exp (\,-\,iC({T}^{2}/{{T}_{0}}^{2}))$$, $$\theta $$ is the angle respect to the x-axis, *C* is the chirp parameter, and $${T}_{0}=\,{T}_{FWHM}/2\,\mathrm{ln}(1+\sqrt{2})$$, where $${T}_{FWHM}$$ is the full width at half-maximum pulse length. The pump wavelength of the input pulse is 1064 nm. Finally, the circularly polarized components of the field envelope in the frequency domain are related to the linear components as $${\tilde{A}}_{x,y}({\rm{\Omega }},z)=[{\tilde{A}}_{1}({\rm{\Omega }},z)\,\pm \,{\tilde{A}}_{2}({\rm{\Omega }},z)]\exp (\,\mp \,i{\rm{\Delta }}{\beta }_{0}/2)/\sqrt{2}$$. Equations (3) and (4) were implemented in Matlab and solved in the frequency domain in the interaction picture method by using a Runge-Kutta (RK4 (3)) scheme for integration of the nonlinear operator^45^. The step size is fixed to 25 µm and the number of points used in the simulation is $${N}_{p}={2}^{16}$$.

**Figure 8:**
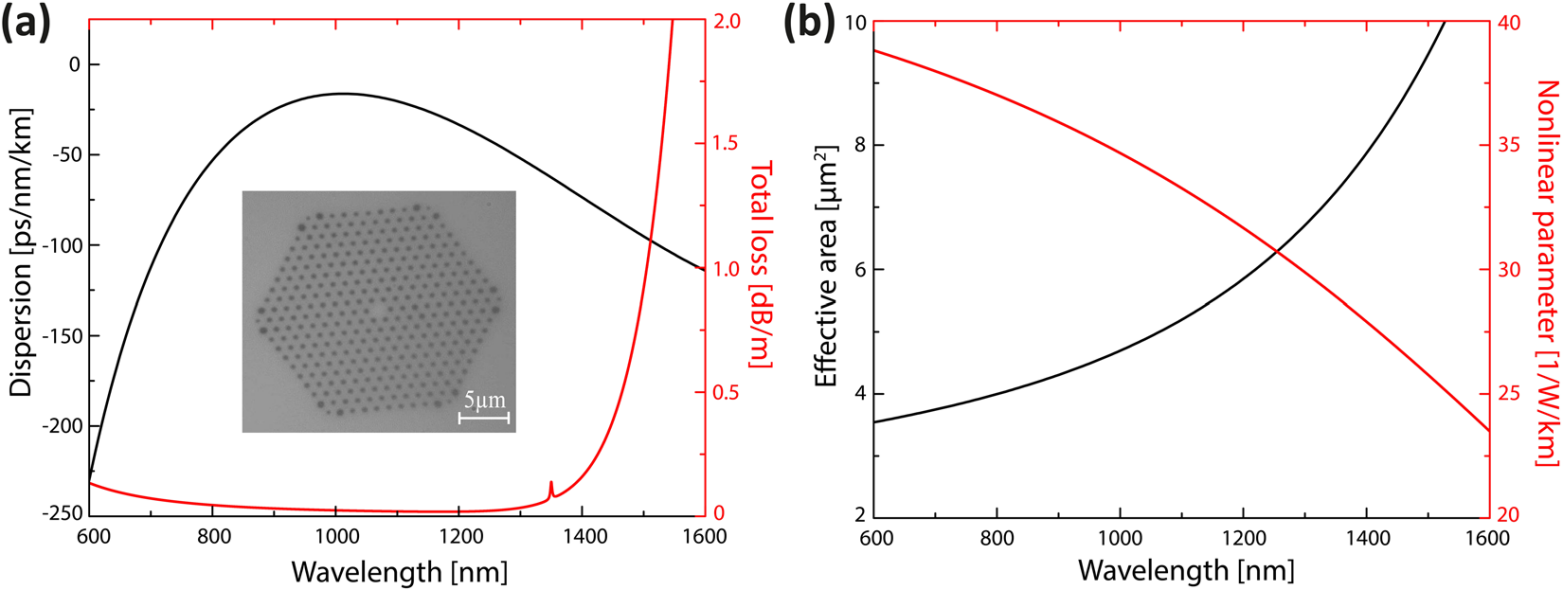
Fiber properties used in the simulations. (**a**) Dispersion profile (black) and total loss (red), including material and confinement loss. (**b**) Effective area (black) and nonlinear parameter (red) for the ANDi fiber NL-1050-NEG-1 (${\rm{\Lambda }}=1.44\,\mu m,$ $d/{\rm{\Lambda }}=0.39$). A cross section of the ANDi fiber is shown in the inset in (**a**).

Simulation parameters were taken from a commercially available ANDi fiber NL-1050-NEG-1^46^ for comparison with the experiments. The cross section of the fiber used is displayed in Fig. 8a. Fiber parameters such as group velocity dispersion, confinement loss and effective area were calculated with COMSOL Multiphysics and are shown in Fig. 8a,b. The frequency dependence of the nonlinear parameter was calculated with equation (6).

Coherence and noise are calculated for all pairs of an ensemble of 20 independent simulations with the same input parameters but different initial quantum noise. Quantum noise is added in both polarizations as one-photon-per-mode, in which a photon with random phase is added to each frequency bin^1,3,47^. The noise is in the frequency domain given by ${\tilde{a}}_{oppm}({{\rm{\Omega }}}_{m})=\sqrt{\hslash ({N}_{p}-1){\mathrm{dT}{\rm{\Omega }}}_{m}}\exp [2\pi {\rm{i}}{\rm{\Phi }}({{\rm{\Omega }}}_{m})]$, where ${\rm{\Phi }}({{\rm{\Omega }}}_{m})$ is the random phase corresponding to a white noise uniformly distributed in the interval [0, 1] in each frequency bin Ω_*m*_. The noise is transformed back to the time domain and added to ${A}_{1,2}(T,0).$ Different noise seeds are used for each polarization^42,48^, and we are assuming no correlation between them. The coherence of the SC ensemble is quantified with the first-order spectral coherence function defined by^1,37^

7 $$|{g}_{12}^{(1)}(\omega )|=|\frac{{\langle {\tilde{A}}_{i}^{\ast }(\omega ){\tilde{A}}_{j}(\omega )\rangle }_{i\ne j}}{\sqrt{\langle {|{\tilde{A}}_{i}(\omega )|}^{2}\rangle \langle {|{\tilde{A}}_{j}(\omega )|}^{2}\rangle }}|=|\frac{2N}{({N}^{2}-N)}\frac{{\sum }_{i\ne j}^{N}{\tilde{A}}_{i}^{\ast }(\omega ){\tilde{A}}_{j}(\omega )}{\sqrt{{\sum }_{i}^{N}{\tilde{A}}_{i}(\omega ){\sum }_{j}^{N}{\tilde{A}}_{j}(\omega )}}|,$$

where $[{\tilde{A}}_{i}(\omega ),\,{\tilde{A}}_{j}(\omega )]$ are independent pairs of SC, being 190 pairs for $N=20$ realizations. The first-order spectral coherence function returns a value between 0 (low coherence) and 1 (high coherence) for each frequency. The spectrally averaged coherence is also calculated as^1^

8 $$\langle |{g}_{12}^{(1)}|\rangle =\frac{{\int }_{0}^{\infty }|{g}_{12}^{(1)}(\omega )|\langle {|\tilde{A}(\omega )|}^{2}\rangle d\omega }{{\int }_{0}^{\infty }\langle {|\tilde{A}(\omega )|}^{2}\rangle d\omega },$$

which gives $0\le \langle |{{g}}_{12}^{(1)}|\rangle \le 1$ for an ensemble of SC. Furthermore, the noise is calculated with the RIN defined as the ratio of the mean to the standard deviation^49^,

9 $${\rm{RIN}}(\omega )=\frac{\sigma (\omega )}{\mu (\omega )}=\frac{{\langle {({|{\tilde{A}}_{i}(\omega )|}^{2}-\mu (\omega ))}^{2}\rangle }^{1/2}}{\langle {|{\tilde{A}}_{i}(\omega )|}^{2}\rangle }=\frac{\sqrt{\frac{1}{N-1}{\sum }_{i}^{N}{({|{\tilde{A}}_{i}(\omega )|}^{2}-\mu (\omega ))}^{2}}}{\frac{1}{N}{\sum }_{i}^{N}{|{\tilde{A}}_{i}(\omega )|}^{2}}.$$

In the same way as with the first-order coherence, the RIN can also be spectrally averaged to yield a single number for a SC ensemble,

10 $$\langle {\rm{RIN}}\rangle =\frac{{\int }_{0}^{\infty }{\rm{RIN}}(\omega )\langle {|\tilde{A}(\omega )|}^{2}\rangle d\omega }{{\int }_{0}^{\infty }\langle {|\tilde{A}(\omega )|}^{2}\rangle d\omega }.$$

### Experimental setup

**Figure 9:**
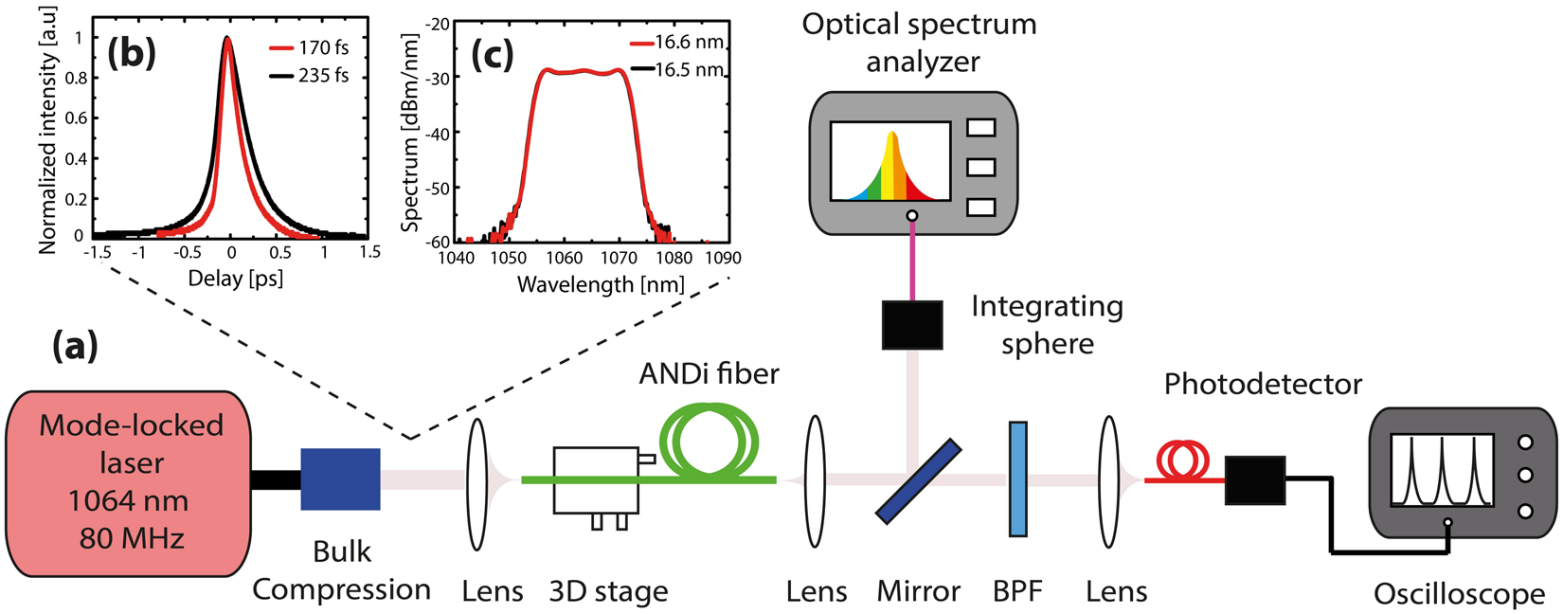
Experimental setup. (**a**) Schematic of the experimental setup for supercontinuum generation and noise measurements; BPF – Bandpass filter. (**b**) Intensity autocorrelation measurement (FWHM) and (**c**) spectrum of the pump laser for two different settings.

The experimental setup is shown in Fig. 9a. A collimated beam from a mode-locked laser with center wavelength at 1064 nm and 80 MHz repetition rate was focused into 0.5 m of ANDi fiber with an aspheric lens. The laser pump is a passively mode-locked laser borrowed from Fianium (FP-1060–5-fs), in which picosecond pulses emitted from the laser are compressed in an external stage with bulk compression. The laser emits a collimated and linearly polarized beam and its output was characterized as shown in Fig. 9b,c. First, the pulse length was measured with an intensity autocorrelator (Femtochrome FR-103HP) and an oscilloscope for two different configurations of the laser (Fig. 9b). The FWHM of the laser was found to be 170 fs and 235 fs, assuming a hyperbolic secant squared shape power profile. Furthermore, the spectra for these two configurations (Fig. 9c) were also measured with an optical spectrum analyzer (ANDO AQ6317B) in order to estimate the linear chirp in the simulations. The measured FWHM bandwidth was 16.6 nm and 15.5 nm for 170 fs and 235 fs, respectively. Nonlinear chirp due to self-phase modulation in the output spectra was also observed because of the amplification stage in the laser.

The SC generated after 0.5 m of ANDi fiber was collimated and measured with the optical spectrum analyzer (OSA) used before. An integrating sphere (~20 dB wavelength independent loss) was used to reduce the power going to the OSA, to avoid damaging the instrument as well as to collect all divergent light. Each spectrum is an average over thousands of pulses due to the slow response of the detector in the OSA. The input power to the ANDi fiber was controlled with neutral density filters from Thorlabs, in order to measure the power dependence of the generated SC and power dependence of the RIN. In these measurements, the input polarization was not adjusted to the slow-axis as in previous studies^34–36^ where they tried to minimize the noise. The noise investigation is here carried out aiming at a practical source where low birefringence fibers are spliced on directly to the pump laser and no extra components are used to match the input polarization to the slow-axis.

To measure the wavelength dependence of the RIN^37,39^, the SC is filtered every 50 nm with bandpass filters of 10 nm bandwidth from Thorlabs. After this, the filtered SC is then detected with two fiber-coupled photodiodes, an InGaAs for longer wavelengths 950–1350 nm (Thorlabs - DET08CFC - 800 to 1700 nm, 5 GHz BW), and a Si for shorter wavelengths 750–900 nm (Thorlabs - DET025AFC - 400 to 1100 nm, 2 GHz BW). Then, the voltage time series are recorded with a fast oscilloscope (Teledyne LeCroy - HDO9404–10 bits resolution, 40 Gs/s, and 4 GHz BW). Around ~16000 pulses are recorded for each 10 nm filtered SC. The power incident on the detector was purposely maintained low to operate in the linear regime, far from saturation. The described technique has been already used to measure the noise in SC sources^50^. To calculate the RIN, we extract the maximum of the pulses from the voltage time series recorded for each 10 nm filtered SC and subtract the noise floor. The histograms obtained for the extracted ~16000 peak values are fitted to a gamma distribution, and the RIN is calculated as the ratio of the standard deviation to the mean of the distribution. Besides the SC noise, the noise of the pump laser was also characterized by measuring the pulse to pulse fluctuations with the photodiode without filter. No filter was used since the width of the pump pulse is close that of the filters. The value obtained was 0.71% (170 fs) and 0.67% (235 fs).

### Data availability

The datasets generated during and/or analyzed during the current study are available from the corresponding author on reasonable request.
